# Development and Laboratory Evaluation of a Cold Mix High-Early-Strength Epoxy Asphalt Concrete for Steel Bridge Deck Pavements

**DOI:** 10.3390/ma14164555

**Published:** 2021-08-13

**Authors:** Yang Liu, Zhendong Qian, Yongning Wang, Yongchao Xue

**Affiliations:** Intelligent Transportation System Research Center, Southeast University, Nanjing 211189, China; qianzd@seu.edu.cn (Z.Q.); seu318222@163.com (Y.W.); xueyc@seu.edu.cn (Y.X.)

**Keywords:** epoxy asphalt concrete, high-early-strength, cold mix, steel bridge deck pavement, curing period

## Abstract

Epoxy asphalt concrete (EAC) is a widely used steel bridge deck pavement (SBDP) material. Due to the curing reaction, the EAC-based material needs a long curing period before opening to traffic, which in an inconvenience in the construction of SBDP. This study developed a cold mix high-early-strength (CHES) epoxy asphalt through the design of a compatilizer and curing agent system. The optimum formula of CHES epoxy asphalt was determined through a series of laboratory tests. By comparison of the performances of CHES EAC and some conventional EACs for SBDP, it was found that the developed CHES epoxy asphalt can significantly reduce the curing period, and the pavement performance of CHES EAC is, overall, excellent for application in SBDP. In addition, the sufficient allowable construction duration shows that the CHES EAC has a good construction workability.

## 1. Introduction

Epoxy asphalt concrete (EAC) is a thermosetting material with desirable mechanical properties and outstanding durability and has been widely used in the steel bridge deck pavement (SBDP) [1,2,3,4]. Differing from the conventional thermoplastic asphalt concrete, the strength of EAC depends upon the curing reaction between the epoxy resin and the curing agent [5,6]. Therefore, the EAC-based pavement needs a long curing period before the bridge can be opened to traffic, which means more construction costs. Cracks and potholes are the most common failure modes of SBDP, and epoxy binders and EAC have been proven to be the excellent repair materials for these failures [6,7,8]. However, the rehabilitated area also needs sufficient curing time to achieve high strength and become well-integrated with the original pavement structure and in reality, the epoxy repair material in most rehabilitation areas does not get fully cured due to the need to resume traffic as soon as possible. Therefore, it is essential to develop a high-early-strength epoxy asphalt material.

Epoxy asphalt is usually mixed at a high temperature that is in the range from 110 °C to 130 °C for warm mix EAC, and in the range from 170 °C to 185 °C for hot mix EAC. The high-temperature paving of EAC is harmful to the environment due to the high energy consumption and emission of toxic fumes and can cause temperature effects on the steel bridge deck as well [9,10,11]. In recent years, the cold mix epoxy asphalt has been developed and applied to some SBDP projects [12,13]. The cold mix epoxy asphalt is not only an eco-friendly material, but also has some other advantages, such as reduction of mixture temperature segregation. Therefore, the development of high-performance cold mix asphalt mixture is a trend in pavement engineering.

The curing reaction can transform the epoxy resin contained in the epoxy asphalt into plastic or rubber by a cross-linking process and subsequently link the molecular chains into a rigid, 3D structure. Meanwhile, the curing reaction is influenced by the temperature and curing time. Therefore, the strength development law of EAC is complicated. Qian et al. developed a curing reaction model to reveal the strength evolution law of EAC [14]. As the components in epoxy asphalt have great influence on its physical and mechanical properties, many researchers have modified the epoxy asphalt by changing its component formulations. Si et al. optimized an epoxy asphalt formulation and developed a cold-mixed epoxy asphalt and cold-mixed epoxy-SBS-modified asphalt [5,15]. Zhang et al. developed a cold-mix high-toughness epoxy resin through introducing a flexible chain into the molecular structure of cured epoxy resin [16]. Fuhaid et al. used epoxidized soybean oil and a biobased curing agent, maleic anhydride, to develop a biobased epoxy asphalt binder for asphalt pavements [17].

The main objective of this study is to develop a cold mix high-early-strength (CHES) epoxy asphalt for SBDP. Through blending the compatilizer and optimizing the curing agent system, a CHES epoxy asphalt was designed and the optimum formula was determined. Meanwhile, the pavement performances of CHES EAC were compared with the conventional EACs in SBDP through a series of laboratory tests, and the feasibility and construction workability of the developed CHES EAC for SBDP was evaluated.

## 2. Materials and Methods

### 2.1. Material Selection

Epoxy asphalt is generally comprised of an epoxy resin, curing agent, and base asphalt. E51 is a common bisphenol-A liquid epoxy resin with the epoxy value of 0.53 mol/100 g and was used to synthesize the CHES epoxy asphalt in this research. The selection and proportion of curing agent have a significant influence on the curing temperature, curing rate, and mechanical properties of EAB. Based on a literature review of recent studies on epoxy asphalt in the chemical industry, a modified aliphatic amine curing agent was selected due to the low curing temperature, fast curing rate and good flexibility for the EAB [18,19,20]. One challenge in formulating CHES epoxy asphalt is the compatibility between the epoxy resin, which is polar, and conventional asphalt, which is nonpolar [21,22,23]. AH70 grade asphalt (70# asphalt) could have better compatibility with epoxy resin than AH90 and AH110 grade asphalts because of the relatively high content of asphaltene and resins. Therefore, the AH70 grade asphalt was selected as the base asphalt for our CHES epoxy asphalt. In addition, a suitable compatilizer that has good compatibility with both of the epoxy resin and base asphalt, is needed to improve the compatibility between epoxy resin and base asphalt and to achieve a homogenous cross-linking between them [24,25]. In this research, a phenolic compatilizer was used as the additive for the CHES epoxy asphalt.

### 2.2. Preparation of CHES Epoxy Asphalt

The CHES epoxy asphalt was composed of component A and component B, where the former is E51 epoxy resin and component B was prepared through mixing the curing agent, base asphalt and compatilizer. The base asphalt was heated to 120 °C for more than 30 min until it was fully fluidized. Subsequently, the base asphalt and the compatilizer were mixed in a three-necked flask, and the mixture was stirred and refluxed at 120 °C for about 1 h. The mixture was cooled to 40 °C, and the curing agent was then added and stirred for 1 h at ambient temperature to obtain component B. Finally, component A and component B were blended in suitable proportions and the blend was stirred for 5 min at ambient temperature to prepare the CHES epoxy asphalt.

### 2.3. Experimental Methods

#### 2.3.1. Segregation Test

The compatibility between asphalt and epoxy resin with different proportion of compatilizer was evaluated by a segregation test. The following test procedure was adopted: (1) heat asphalt at 120 °C for 30 min, (2) mix the compatilizer with the fluidized asphalt, (3) cool the mixture of asphalt and compatilizer to 40 °C, (4) add the epoxy resin to the mixture and stir evenly, (5) pour the mixture into a glass tube with the lubricant coated onto the inner wall, (6) let the glass tube stand for 48 h, (7) freeze in the refrigerator for 4 h and subsequently take out the mixture specimen and (8) measure the softening points of the upper and lower parts of the asphalt-resin specimen. The softening point difference can reflect the compatibility between asphalt and epoxy resin, and the optimum proportion of compatilizer was determined to have a softening point difference of less than 1 °C.

#### 2.3.2. Tensile Test

**Figure 1:**
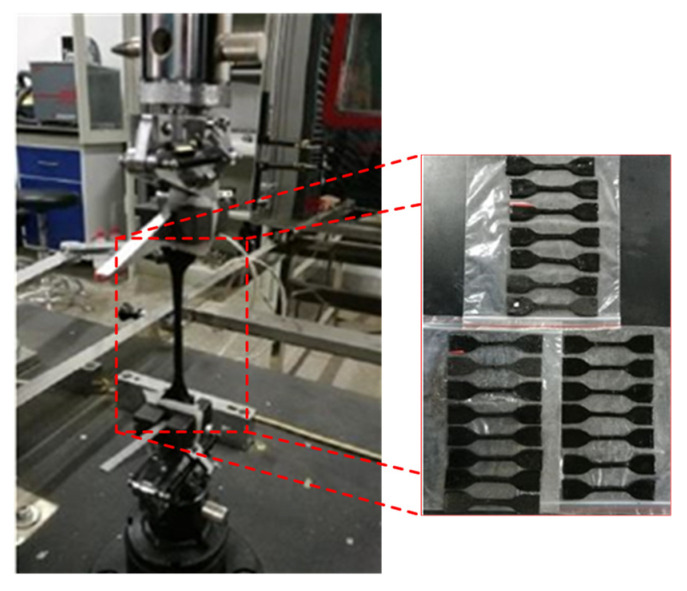
Dumbbell-shaped specimens and tensile test.

The tensile strength and elongation at break are important mechanical indexes for an epoxy asphalt, as they can reflect the strength and deformation properties. The tensile strength and elongation at break of CHES epoxy asphalt was measured through a tensile test, following a standard test procedure in ASTM D638 [26], as shown in Figure 1. Dumbbell-shaped specimens of CHES epoxy asphalt were prepared, following the specimen dimension of type I as defined in ASTM D638. The tensile test was conducted on a direct tensile tester (5525-type machine produced by INSTRON Co., Ltd., Norwood, MA, USA) at 25 °C with the testing rate of 500 mm/min.

#### 2.3.3. Rotational Viscosity Test

Due to its unique thermosetting features, the viscosity of epoxy asphalt increases with the curing reaction, and the increasing rate of viscosity has a great influence on the workability and curing period of epoxy asphalt. The viscosity of CHES epoxy asphalt was measured by a Brookfield viscometer (DV-II, Brookfield Engineering Inc., Middleboro, MA, USA), following a standard test procedure described in AASHTO T316 [27]. The CHES epoxy asphalt was prepared and immediately poured into the viscometer container at 25 °C. The viscosity data were subsequently recorded every 10 min for 120 min.

#### 2.3.4. Marshall Stability Test

**Figure 2:**
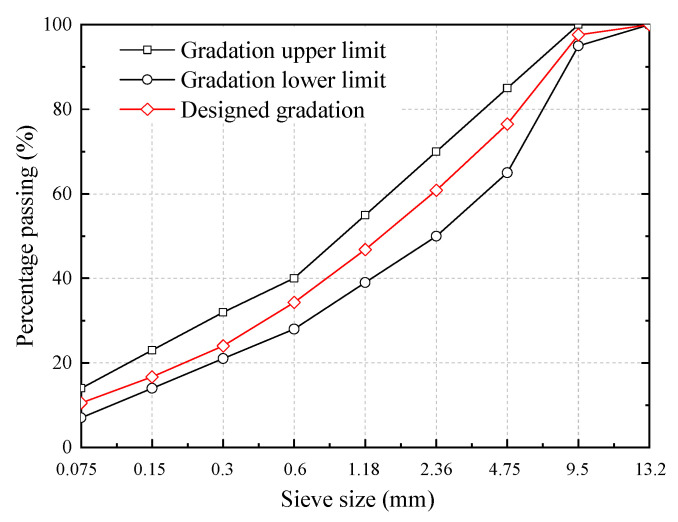
Gradation of CHES epoxy asphalt mixture.

After the laboratory evaluation of the CHES epoxy asphalt, its effectiveness also needs to be evaluated in the context of asphalt mixture performance. The pavement-related properties of CHES epoxy asphalt mixture were evaluated by the Marshall stability test, following a standard test procedure in AASHTO T245 [28]. Basalt aggregates and limestone powder sourced from a quarry located in Jurong (China), were used preparingfor CHES epoxy asphalt mixtures. A dense gradation with the nominal maximum aggregate size of 9.5mm (as shown in Figure 2), which has been widely used for the EAC pavement in China, was selected to achieve mix designs with good impermeability and durability, and the content of CHES epoxy asphalt was determined as 8.5% (by mass of aggregate) based on the Marshall mixture design procedure.

Since the specimens of CHES epoxy asphalt mixture would gain the strength and stiffness over time due to binder curing, the Marshall stability and flow values of CHES epoxy asphalt mixture after different curing periods were measured, including curing periods of 8, 10, 12, 16, 20 and 24 h. The steps to prepare and test the CHES epoxy asphalt mixture specimens are as follows:

(1) The CHES epoxy asphalt was mixed with aggregates at ambient temperature to form a mixture.
(2) The mixture was compacted in a Marshall compactor with 75 blows on each side, and the Marshall specimens were extracted from the mold and kept at the room temperature (25 °C) for different curing periods. In addition, the volume parameters of the molded mixture were measured.
(3) The specimens were immersed in the 60 °C constant-temperature water for 0.5 h, and the Marshall stability and flow values were measured subsequently.

#### 2.3.5. Pavement Performance Test

**Figure 3:**
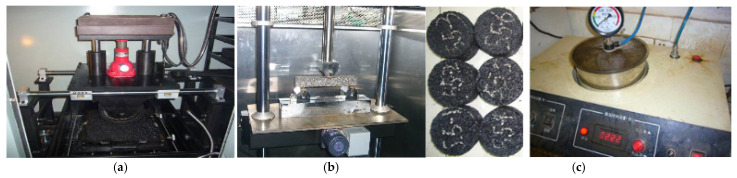
Pavement performance test for EAC (**a**) Wheel tracking test; (**b**) Bending beam test; (**c**) The soaked Marshall test and indirect tensile strength ratio test.

The CHES EAC should have adequate high-temperature rutting resistance, low-temperature cracking resistance, and moisture susceptibility for its application to the steel bridge deck. The wheel tracking test and bending beam test were respectively used to evaluate the high-temperature rutting resistance and low-temperature cracking resistance, and the detailed test procedures have been described in the authors’ published literature [29]. The moisture susceptibility could be evaluated by the soaked Marshall test and indirect tensile strength ratio (TSR) test, following procedures in Chinese test specifications JTG E20-2011 [30]. The soaked Marshall test and indirect tensile strength ratio test were conducted on the Marshall specimens, and the TSR and residual Marshall stability were calculated to evaluate the moisture susceptibility of EAC. The pavement performance tests are shown in Figure 3.

## 3. Results and Discussions

### 3.1. Formula Design of CHES Epoxy Asphalt

The proportions of epoxy resin, curing agent, base asphalt, and compatilizer have a great influence on the strength, curing period, allowable construction duration, and viscosity properties of epoxy asphalt. The formula of CHES epoxy asphalt was determined based on three principles. Firstly, the compatibility between asphalt and epoxy resin should be evaluated to determine the optimum proportion of compatilizer. Secondly, the CHES epoxy asphalt should have an adequate allowable construction duration, which means the initial curing rate should not be fast. In general, the time for viscosity of epoxy asphalt increasing to 3 Pa·s is used to evaluate the allowable construction duration of epoxy asphalt [31]. Thirdly, the CHES epoxy asphalt should achieve ultimate strength in a short period at ambient temperature.

#### 3.1.1. Optimum Proportion Determination of Compatilizer

The segregation test results are listed in Table 1. When the proportion of compatilizer (by mass of epoxy resin) exceeded 20%, the softening point difference value of the lower part and upper part of asphalt-resin specimen was less than 1 °C, and it was regarded that the asphalt and epoxy resin have a good compatibility. Given that too much compatilizer may have an adverse influence on the cross-linking behavior of epoxy asphalt, the proportion of compatilizer was set at 20% (by mass of epoxy resin).

#### 3.1.2. Curing Agent Design

In the curing agent system of the CHES epoxy asphalt, a modified aliphatic amine curing agent was used as the base curing agent to ensure the mechanical properties of epoxy asphalt, and a synergistic curing agent produced by Jiangsu Zhongyitong New Road Materials Co., Ltd. (Jiangyin City, China), was used to shorten the time for curing reaction of the epoxy asphalt. In this study, five types of modified aliphatic amine curing agents (named curing agents A1~A5) were used to prepare epoxy asphalts, and the base curing agent for CHES epoxy asphalt was selected through comparing the properties of different samples. When preparing the epoxy asphalt, the mass proportion of modified aliphatic amine curing agent, E51 epoxy resin, compatilizer, and 70# asphalt was 1:1:0.2:2.2. For each modified aliphatic amine curing agent, 10 groups of epoxy asphalt samples were prepared for testing. One group of samples was used to measure the viscosity variation with time at 25 °C, and the allowable construction duration (i.e., the time for viscosity increasing to 3Pa·s) was determined. The other samples were used to conduct tensile tests in which the tensile strength and elongation at break of the epoxy asphalts were measured. To determine the fully-cured time of the different epoxy asphalts (i.e., the curing time for epoxy asphalt reaching the ultimate strength), the nine groups of samples were cured at 25℃ with different curing times (12, 16, 20, 24, 36, 48, 60, 72 and 96 h, respectively), and the tensile strength varied with the curing time were measured. The test results of rotational viscosity test and tensile test are given in Table 2.

As shown in Table 2, the elongation at break of epoxy asphalt with A3, A4 and A5 curing agent is lower than 80%, meaning a weak toughness. While the epoxy asphalt with A1 and A2 curing agent after fully cured has large tensile strength and has good toughness as well. Compared with the A1 curing agent and A2 curing agent, the epoxy asphalt with A1 curing agent has a longer allowable construction duration and shorter fully cured time. Therefore, the A1 curing agent is selected as the base curing agent for the CHES epoxy asphalt.

The synergistic curing agent (named SY curing agent) was used to further shorten the fully cured time of epoxy asphalt, and the test results of epoxy asphalt with different mass proportions of the base curing agent and synergistic curing agent are listed in Table 3. It could be found that the ultimate tensile strength of epoxy asphalt and allowable construction duration reduce with the increasing proportion of SY curing agent, but the toughness increases too. The field construction experiences of SBDP show that it is more conductive to the construction organizations if the allowable construction duration exceeds 100 min. In addition, according to the requirements of epoxy asphalt binder for steel bridge deck pavement laid out in the Standard JTG/T3364-02 [32], the tensile strength should be larger than 2.0 MPa, and the elongation at break should be larger than 100%. Therefore, the best mass proportion of A1 and SY curing agent is suggested to be 9:1, and in this situation that the fully-cured time is reduced 45% compared to the epoxy asphalt without the SY curing agent.

#### 3.1.3. Optimum Formula Determination of CHES Epoxy Asphalt

In this section, the best proportion of epoxy resin, curing agent, and 70# asphalt was determined. Based on the previous studies and for cost reasons [33,34], 16 groups of formula schemes were preset to conduct the tensile test. The test results are listed in Table 4.

**Figure 4:**
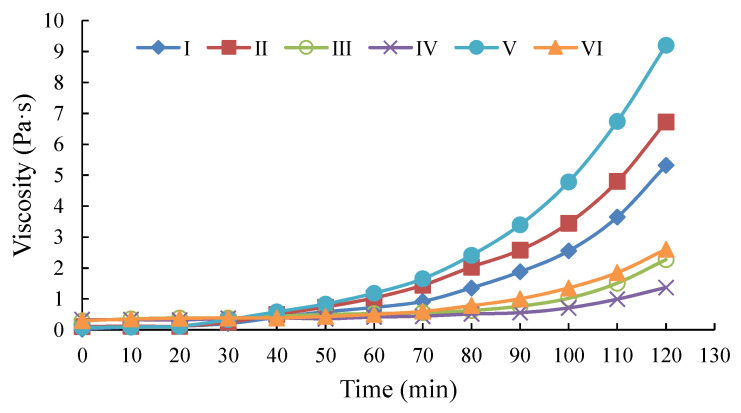
Rotational viscosity test results for epoxy asphalts with different formula.

With the increase of curing agent, the degree of curing reaction between the epoxy resin and curing agent improves, resulting in an increase of tensile strength but a decrease of elongation at break (i.e., decrease of toughness). However, when the mass ratio of curing agent to epoxy resin exceeds 0.8, the redundant curing agent could reduce the cross-linking degree of the cured product, resulting in a reduction of tensile strength. As the proportion of 70# asphalt increases, the tensile strength of epoxy asphalt decreases, and the toughness increases. However, when the proportion of 70# asphalt exceeds 80%, the curing reaction degree of epoxy asphalt is very low, and the tensile strength is too small. Given that the tensile strength of epoxy asphalt for SBDP should be larger than 2.0 MPa, and the elongation at break should be larger than 100%, some formula schemes in Table 4 should be eliminated, and the remaining six formula schemes were numbered and a rotational viscosity test was conducted for further comparison. The numbers of the six formula schemes are given in Table 5, and the rotational viscosity test results are given in Figure 4. Given the allowable construction duration (i.e., the time for viscosity increasing to 3 Pa·s) should be larger than 100 min, Formula III, Formula IV, and Formula VI are selected.

To determine the optimum formula of CHES epoxy asphalt, the epoxy asphalts with Formula III, Formula IV, and Formula VI were used to prepare the EAC and conduct the Marshall stability test. The test results are given in Table 6. It is clear that the strength increase of EAC with Formula III is faster than with the other two formulas, therefore, Formula III is selected as the optimum formula for CHES epoxy asphalt, and the proportion of epoxy resin, curing agent, compatilizer, and 70# asphalt is set at 1:0.8:0.2:3.

### 3.2. Strength Comparison of CHES EAC with Other EACs

As of today, the EACs applied to SBDP contain hot mix EAC, warm mix EAC, and cold mix EAC. The purpose of the design of CHES epoxy asphalt in this study is to reduce the curing period for EAC while reaching the ultimate strength. In this section, the strength increase trend of CHES EAC was compared with the conventional hot mix EAC, warm mix EAC, and cold mix EAC. In this study, the hot mix epoxy asphalt tested is the KD-BEP epoxy asphalt imported from Japan, the warm mix epoxy asphalt was a 2910-type epoxy asphalt produced by Jurong Ningwu New Material Co., Ltd. (Jurong City, China) and the cold mix epoxy asphalt was produced by Jiangsu Zhongyitong New Road Materials Co., Ltd. The basic properties of these epoxy asphalts are listed in Table 7. The aggregate used in EAC is basalt aggregate and limestone powder sourced from a basalt quarry located in Jurong (China), and its density is 2.65 g/cm^3^. The aggregate gradation of EAC is shown in Figure 2. Based on the Marshall mixture design procedure [35,36,37,38], the optimum asphalt-aggregate ratio for CHES EAC, KD-BEP EAC, V-type EAC, and cold mix EAC are 8.3%, 6.5%, 6.5%, and 8.5%, respectively.

**Figure 5:**
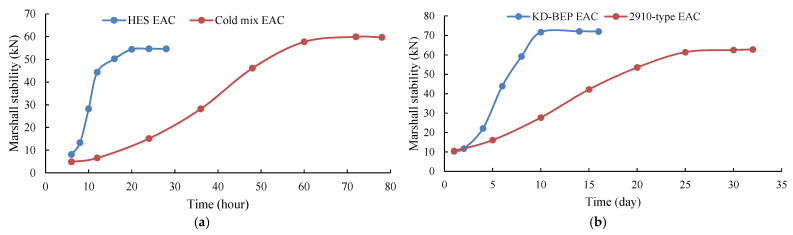
Strength increasing trends of different EACs with time (**a**) CHES EAC and cold mix EAC; (**b**) KD-BEP EAC and 2910-type EAC.

Through Marshall stability tests, the strength variations of different EACs with time were measured, and the results are given in Figure 5. It could be found that the curing time for CHES EAC to attain its ultimate strength at 25 °C is about 20 h, while that for cold mix epoxy asphalt, KD-BEP epoxy asphalt, and 2910-type epoxy asphalt the times are about 72 h, 10 days and 30 days, respectively. Accordingly, the developed CHES epoxy asphalt can significantly reduce the curing period for EAC while still reaching the ultimate strength. The ultimate strengths of CHES EAC, cold mix EAC, KD-BEP EAC, and 2910-type EAC are 55kN, 59kN, 72kN, and 62kN, respectively. Although the ultimate strength of CHES EAC is little lower than that of the conventional EACs, it greatly exceeds the strength requirement (40 kN) of EAC on steel bridge decks [32].

### 3.3. Pavement Performances of CHES EAC

The high-temperature rutting resistance, low-temperature cracking resistance and moisture susceptibility of CHES EAC were evaluated and compared with the conventional EACs. The results are given in Table 8. The high-temperature rutting resistance of CHES EAC is weaker than those of KD-BEP EAC and 2910-type EAC, but little higher than that of the cold mix EAC. The low-temperature cracking resistance of CHES EAC is a little weaker than that of the cold mix EAC, but higher than those of the KD-BEP EAC and 2910-type EAC. In addition, the CHES EAC has the best moisture susceptibility among all the EACs. Accordingly, the pavement performance of CHES EAC is, overall, excellent for application in SBDP.

### 3.4. Construction Workability of CHES EAC

In the production of CHES EAC, the epoxy asphalt is mixed with the aggregate at ambient temperature. In a normal humid environment, the aggregate is subject to moisture. The moisture will be retained in the CHES EAC during its production, which may affect the performance of CHES EAC. Therefore, the influence of moisture content of the aggregate on the performance of CHES EAC should be studied. In addition, the allowable construction duration is a key construction factor for EAC. The allowable construction duration for CHES EAC at different ambient temperatures should be clear to evaluate the construction workability of CHES EAC.

#### 3.4.1. Effect of Moisture in the Aggregate

**Figure 6:**
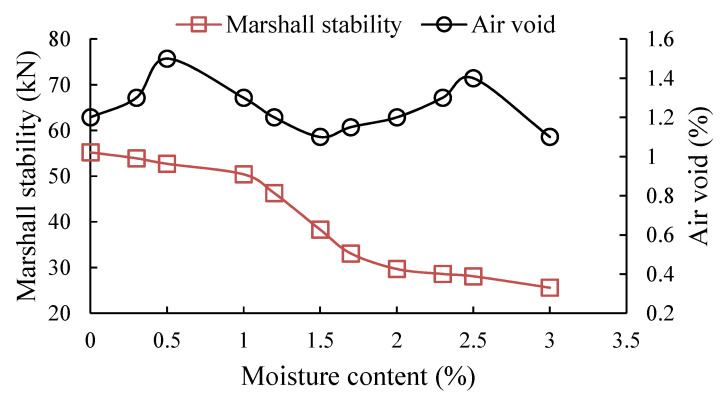
Relationship between aggregates’ moisture content and the performance of CHES EAC.

**Scheme 1:**
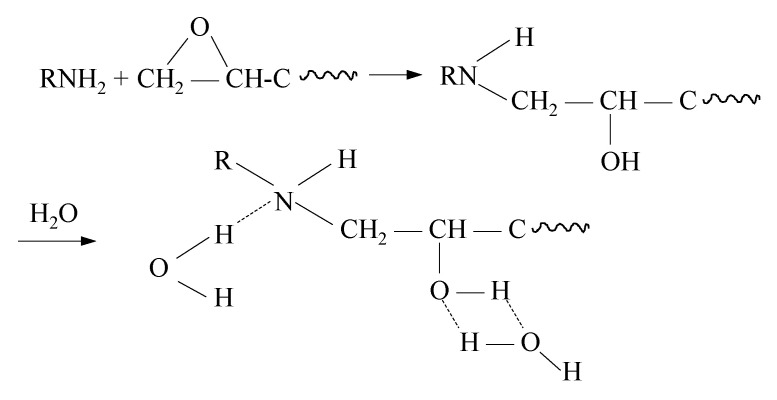
Hydrogen bonding reaction of active groups.

In this study, the aggregates were treated in a humidity chamber to simulate the aggregate in different humid environments. After the treatment process, the moisture content of aggregate was measured, and the aggregates were used to fabricate the CHES EAC. Through the Marshall stability test, the relationship between the aggregates’ moisture content with the performance of CHES EAC was obtained, as shown in Figure 6. It could be found that the air voids of CHES EAC under different moisture contents experience a fluctuation variation. As the design air void for the EAC pavement on the steel bridge deck is 1.0%~3.0% [32], the effect of the aggregates’ moisture content on the air void of CHES EAC could be neglected. However, the Marshall stability of CHES EAC decreases with the increasing of moisture content in the aggregate. When the moisture content increases from 0% to 1%, the Marshall stability decreases slowly, and the Marshall stability of CHES EAC is larger than 50 kN. When the moisture content exceeds 1%, the Marshall stability decreases fast. The Marshall stability is less than 40 kN if the moisture content exceeds 1.5%. As the Marshall stability requirement of EAC for steel bridge should be larger than 40 kN according to the Chinese standard [32], then the moisture content of aggregate for CHES EAC should not exceed 1.5%. With the moisture content increases to more than 2%, the decrease of Marshall stability gets slow again. The variation trend of Marshall stability with the moisture content could be explained by the effect of water molecules on the epoxy asphalt curing reaction. The curing agent for CHES EAC in this study is the modified amine curing agent, and the ring-opening reaction between the curing agent and epoxy resin produces amine active groups. However, the water molecules in the aggregates will go into the epoxy system when the aggregates are mixed with CHES epoxy asphalt, and these amine active groups are likely to undergo hydrogen bonding reactions with the water molecules, as shown in Scheme 1. The hydrogen bonding reaction is not conductive to the formation of 3D cross-linking structure in the epoxy system. This is why the Marshall stability decreases with the moisture content. In addition, when the moisture content is high, the water gradually intrudes into the contact interface between the epoxy asphalt and aggregate, resulting in the reduction of adhesion between the epoxy asphalt and aggregate. This is why a rapid decrease of Marshall stability appears when the moisture content increases to 1%. As the moisture content continues to increase, the water film between epoxy asphalt and aggregate becomes close to saturation, and the Marshall stability tends to stabilize.

In view of the above results and discussions, it is suggested that the moisture content of aggregate for CHES EAC should be less than 1%. If the moisture content of aggregate is high, a drying treatment should be adopted for the aggregate before producing the CHES EAC.

#### 3.4.2. Allowable Construction Duration Determination

The curing reaction begins once the two components of epoxy asphalt are mixed, therefore, the mixture’s strength is increasing during the mixture transportation, and mixture paving process. The allowable construction duration was defined as the time from the completion of mixture production to the initiation of mixture compaction. The strength of CHES EAC before compaction should not be too high to ensure its compaction quality. Therefore, the allowable construction duration of CHES EAC at different ambient temperatures should be evaluated.

**Figure 7:**
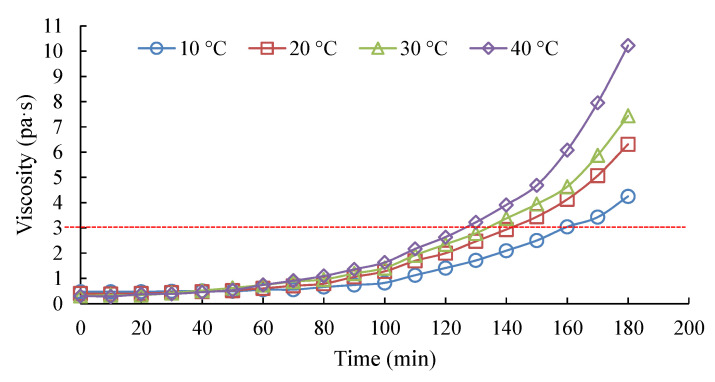
Viscosity variations of CHES epoxy asphalt with time.

**Figure 8:**
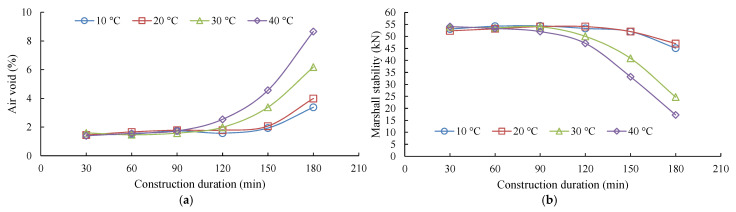
Performance variations of CHES EAC after different construction durations (**a**) Air void; (**b**) Marshall stability.

In this study, the ambient temperatures were selected as 10 °C, 20 °C, 30 °C, and 40 °C. The allowable construction duration was determined through the rotational viscosity test and Marshall stability test. The viscosity variations of CHES epoxy asphalt with time at different temperatures are given in Figure 7. As mentioned before, the time for the viscosity of epoxy asphalt to increase to 3 Pa·s is usually used to evaluate the allowable construction duration of EAC. It can be seen from Figure 7 that the times needed for the viscosity of CHES epoxy asphalt to increase to 3 Pa·s under four temperatures are all longer than 120 min. Then, the allowable construction durations for CHES EAC at different ambient temperatures were further clarified through the Marshall stability test. After the CHES epoxy asphalt mixture was produced, the mixture was divided into groups and kept at certain temperature for a certain time, which was used to simulate the construction duration of mixture transportation and mixture paving during the field construction. The construction duration was selected as 30, 60, 90, 120, 150 and 180 min, respectively. After that, the mixture was taken out and molded to the Marshall specimens by the Marshall compactor. The variations of air void and Marshall stability of CHES EAC after different construction durations were measured, and the results are given in Figure 8. It can be found that when the construction duration increases (i.e., the mixture transportation and mixture paving during the field construction of SBDP), the air void of CHES EAC is increasing but the Marshall stability is decreasing. It is indicated that the CHES EAC is hard to compact if the construction duration gets longer. The allowable construction duration for CHES EAC should ensure its air void and Marshall stability meet the requirements for SBDP. As mentioned before, the air void of EAC should be in the range of 1.0%~3.0%, and the Marshall stability of EAC should be larger than 40kN. According to results in Figure 8, the allowable construction duration of CHES EAC at the ambient temperature of 10 °C and 20 °C exceeds 150 min, and the allowable construction duration at the ambient temperature of 30 °C and 40 °C is about 120 min. The sufficient allowable construction duration shows that the developed CHES EAC has good construction workability.

## 4. Conclusions

This study developed a CHES epoxy asphalt for SBDP. The optimum formula of CHES epoxy asphalt was determined and the performances of different CHES EAC samples were evaluated through a series of laboratory tests. The results of this study can be summarized as follows:

(1) A new CHES epoxy asphalt was developed through blending a compatilizer and optimizing the curing agent system, and the optimum proportion of epoxy resin, curing agent, compatilizer, and 70# asphalt in the CHES epoxy asphalt is 1:0.8:0.2:3.
(2) The developed CHES epoxy asphalt can significantly reduce the curing period for EAC, and the curing time for CHES EAC to reach its ultimate strength at 25 °C is about 20 h.
(3) The CHES EAC has good high-temperature rutting resistance, low-temperature cracking resistance, and moisture susceptibility, and its pavement performance is, overall, excellent for application in SBDP.
(4) The moisture in the aggregate has an adverse effect on the Marshall stability of CHES EAC. It is suggested that the moisture content of aggregate for CHES EAC should be less than 1%. If the moisture content of aggregate is high, a drying treatment should be applied to the aggregate before producing the CHES EAC.
(5) The allowable construction duration of CHES EAC at the ambient temperatures of 10 °C and 20 °C exceeds 150 min, and the allowable construction duration at the ambient temperature of 30 °C and 40 °C is about 120 min.

## Figures and Tables

**Table 1:** Segregation test results.

The Proportion of Compatilizer (by Mass of Epoxy Resin) (%)	Softening Point (°C)		
	Lower Part of Asphalt-Resin Specimen	Upper Part of Asphalt-Resin Specimen	Difference Value
0	45.2	27.3	17.9
10	35.5	33.3	2.2
15	34.9	33.6	1.3
20	34.3	33.9	0.4
30	33.9	33.3	0.6
40	33.6	32.8	0.8
50	32.7	31.7	1.0

**Table 2:** Test results of epoxy asphalt with different modified aliphatic amine curing agents.

Curing Agent Types	Ultimate Tensile Strength (MPa)	Elongation at Break (%)	Allowable Construction Duration (25 °C, min)	Fully Cured Time (h)
A1	5.1	120	120	36
A2	5.7	104	40	48
A3	4.1	75	60	60
A4	2.7	67	30	24
A5	8.1	34	20	72

**Table 3:** Test results of epoxy asphalt with different proportions of SY curing agent.

Mass Proportion of A1 and SY Curing Agent	Ultimate Tensile Strength (MPa)	Elongation at Break (%)	Allowable Construction Duration (25 °C, min)	Fully Cured Time (h)
5:5	1.7	320	30	12
6:4	2.1	284	50	16
7:3	2.6	256	60	16
8:2	3.1	184	80	20
9:1	3.8	146	100	20
10:0	5.1	120	120	36

**Table 4:** Tensile test results of epoxy asphalt with different formula.

Property	Mass Ratio (Epoxy Resin: Curing Agent)	Mass Ratio of 70# Asphalt to Epoxy Asphalt (%)			
		50	60	70	80
Tensile strength (MPa)	1:0.4	1.89	1.32	1.05	–
	1:0.6	2.74	1.84	1.17	–
	1:0.8	4.12	3.42	2.36	–
	1:1	3.80	2.56	1.93	1.26
Elongation at break (%)	1:0.4	266	289	356	–
	1:0.6	187	274	322	–
	1:0.8	103	174	235	–
	1:1	146	214	254	303

**Table 5:** Numbers of formula schemes for epoxy asphalt.

Formula No.	Component Proportion of Epoxy Asphalt	
	Mass Ratio (Epoxy Resin: Curing Agent)	Mass Ratio of 70# Asphalt to Epoxy Asphalt (%)
I	1:0.6	50
II	1:0.8	50
III	1:0.8	60
IV	1:0.8	70
V	1:1	50
VI	1:1	60

**Table 6:** Results of Marshall stability test for different EAC.

Property	Formula No.	Curing Period					
		8 h	10 h	12 h	16 h	20 h	24 h
Marshall stability (kN)	III	13.26	28.20	44.32	50.23	54.4	54.64
	IV	12.8	24.21	40.27	42.31	44.76	44.86
	VI	10.14	20.32	42.10	47.43	48.46	48.64

**Table 7:** Basic properties of epoxy asphalts.

Property	Test Value			
	CHES Epoxy Asphalt	KD-BEP Epoxy Asphalt	2910-Type Epoxy Asphalt	Cold Mix Epoxy Asphalt
Tensile strength (MPa)	3.42	2.6	3.86	4.62
Elongation at break (%)	174	226	244	88
Water absorption rate (%, 7 days, 20 °C)	0.12	0.15	0.05	0.13
Time until viscosity increased to 1Pa·s (min)	80	>120	59	67

**Table 8:** Pavement performances of different EACs.

Performance	Test Value				Criterions [32]
	CHES EAC	Cold Mix EAC	KD-BEP EAC	2910-Type EAC	
Dynamic stability (cycle/mm, 70 °C)	26,400	24,020	32,373	29,523	≥6000
Flexural strength (MPa, −10 °C)	19.22	20.52	18.29	18.80	–
Flexural stiffness (MPa, −10 °C)	4953	4869	5685	5275	–
Flexural strain at failure (με, −10 °C)	3880	4214	3218	3564	≥3000
Residual Marshall stability (%)	93.5	91.3	91.1	90.2	≥85
TSR (%)	89.1	86.3	86.5	85.6	≥80

## Data Availability

Data is available upon request from authors.
